# Pneumothorax spontané secondaire post opératoire compliquant une paralysie récurrentielle

**DOI:** 10.11604/pamj.2014.18.208.4843

**Published:** 2014-07-07

**Authors:** Toufik Joulali, Ali Derkaou, Abdelkarim Shimi, Mohammed Khatouf

**Affiliations:** 1 Service de Réanimation Polyvalente A1, CHU Hassan II, Fès, Maroc

**Keywords:** Pneumothorax spontané, paralysie récurrentielle, métastases pulmonaires, tumeurs laryngées, spontaneous pneumothorax, laryngeal paralysis, lung metastases, laryngeal tumors

## Abstract

Le Pneumothorax spontané est défini par un épanchement gazeux de la grande cavité pleurale en dehors de tout traumatisme ou manipulation instrumentale. Son incidence est estimée à 28/100000 pour les hommes et 6/100000 pour les femmes. Les étiologies sont dominées par la broncho-pneumopathies chroniques et obstructives. Le tableau clinique est souvent grave d'emblé nécessitant une exsufflation à l'aiguille et/ou un drainage thoracique. Les récidives sont assez fréquentes et la mortalité reste assez élevée en comparaison avec les pneumothorax post traumatique ou les pneumothorax primaires. Nous rapportons le cas d'une patiente présentant en post opératoire un pneumothorax spontané sur un poumon métastatique et compliquant une paralysie récurrentielle.

## Introduction

Le pneumothorax spontané est une pathologie assez fréquente aux urgences. Il est qualifié de secondaire quand il survient sur un poumon pathologique et la morbi-mortalité dans ce cas devient non négligeable en raison de la fonction pulmonaire déjà compromise chez les patients. Nous présentons le cas clinique d'une patiente suivie pour une tumeur laryngée localement avancée avec des métastases pulmonaires, opérée sous anesthésie générale pour une biopsie profonde de la tumeur et présentant en post-opératoire un pneumothorax spontané secondaire.

## Patient et observation

**Figure 1:**
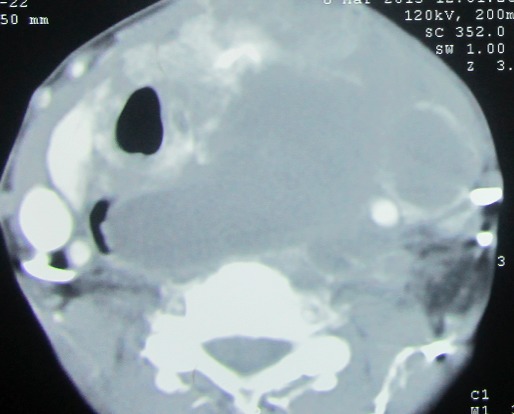
Coupe scannographique objectivant une tumeur laryngée localement avancée

**Figure 2:**
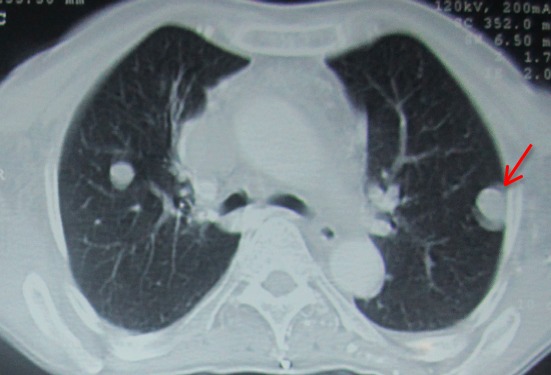
Coupe scannographique à fenêtre parenchymateuse objectivant les métastases pulmonaires bilatérales

**Figure 3:**
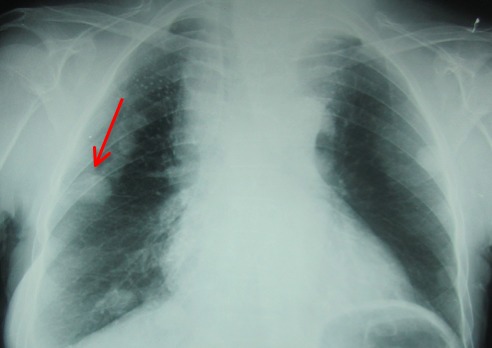
Radiographie du thorax en pré-opératoire montrant les métastases pulmonaires

**Figure 4:**
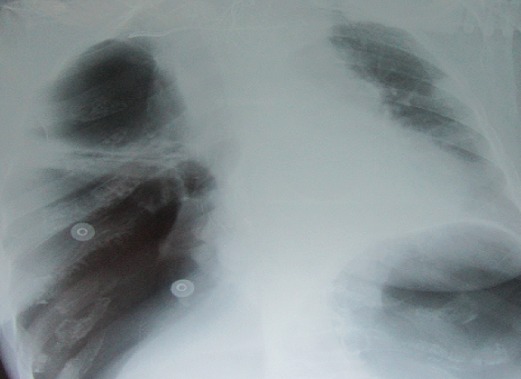
Radiographie du thorax montant un pneumothorax droit de grande abondance refoulant le poumon au niveau médiastinal

**Figure 5:**
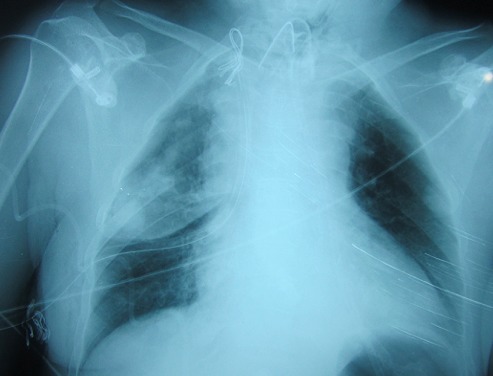
Radiographie de thorax en post-drainage montrant le retour du poumon à la paroi

Madame H.R âgée de 59 ans, suivie en oto-rhino-laryngologie pour une tumeur laryngée localement avancée (Figure 1) avec une atteinte récurrentielle homolatérale et métastases pulmonaires (Figure 2). La patiente a été admise au bloc opératoire pour une biopsie cervicale, faite sous anesthésie générale avec une intubation facile par sonde N°7 et une induction anesthésique faite par du propofol, fentanyl et vécuronum après un monitorage standard normal. Le geste a consisté à une cervicotomie antérieure haute avec nécrosectomie et biopsie de la tumeur pour une étude anatomo-pathologique. Après 5min de son extubation et son admission à la SSPI, la patiente a présenté d'une façon brutale suite à un accès de toux, une détresse respiratoire avec désaturation, cyanose généralisée, sueurs et une bradycardie extrême indiquant sa ré-intubation avec au monitorage du respirateurs des pressions en plateaux à 55 mm h2O, un tympanisme au niveau de l'hémithorax droit avec un silence auscultatoire et un emphysème sous cutané faisant évoquer un pneumothorax confirmé par la radiographie standard (Figure 3, Figure 4). La patiente a été exsufflée en urgence à l'aiguille puis drainée au service de réanimation avec une stabilité clinique de la patiente et un retour du poumon à la paroi sur la radiographie de contrôle (Figure 5). Vu l’état pulmonaire de la patiente une trachéotomie d'emblée pour sevrage respiratoire a été réalisée avec une évolution favorable et son transfert au service d'ORL pour complément de prise en charge.

## Discussion

Le pneumothorax spontané est défini par la présence d'air dans la cavité pleurale en dehors de tout traumatisme ou de manœuvre instrumentale. Il peut survenir sur un poumon sain et dit pneumothorax spontané primaire (PSP) ou sur un poumon pathologique et il est dit pneumothorax spontané secondaire (PSS) [1]. L'incidence globale du PSS est estimée entre 7,4 et 28\100000 pour les hommes et entre 1,2 et 6\100000 pour les femmes. Il touche toutes les tranches d’âge de la pédiatrie à la gériatrie avec une prévalence maximale chez les individus jeunes. [2]. les récidives sont assez fréquentes: 25% à 2ans et 54% à 4ans [3].

La pathogénie exacte de la survenance spontanée d'une communication entre les espaces alvéolaires et la plèvre reste inconnue et repose sur des hypothèses controversées dans la littérature [4]. En temps normal le poumon adhère à la paroi thoracique grâce à la pression négative intrapleurale (-5 cm d'eau) liée aux forces de rétraction élastique s'exerçant sur le poumon et l’élasticité de la cage thoracique [5]. Le pneumothorax primaire peu être expliqué par la présence d'une communication entre les alvéoles et la grande cavité pleurale. Dans la majorité des cas c'est une rupture de blebs ou de bulles d'emphysème suite à des variations de pression atmosphérique [6].

Pour le PSS une multitude de troubles respiratoires ont été décrits comme causes. En tête de fil viennent la maladie pulmonaire obstructive chronique et la maladie pulmonaire interstitielle [7]. Les autres causes décrites sont la fibrose kystique, la tuberculose, le cancer du poumon et les lésions secondaires qui constituent une zone de faiblesse pulmonaire et qui peuvent, suite à une élévation des pressions alvéolaires, constituer une fistule alvéolo-pleurale responsable d'un pneumothorax [6, 7]. C’était le cas chez notre patiente porteuse de métastases pulmonaires et en post- extubation suite à un effort de toux sur une glotte fermée; causée par la paralysie récurrentielle; elle a présenté un pneumothorax suffocant.

En raison de la fonction pulmonaire déjà compromise chez ces patients, le pneumothorax spontané secondaire présente souvent une maladie potentiellement mortelle exigeant une action immédiate [5]. La dyspnée est le maître symptôme associé à une douleur thoracique, cyanose, hypoxémie et hypercapnie entraînant une insuffisance respiratoire aigue. Le tableau clinique peut être plus alarmant et signalant un pneumothorax sous tension se manifestant par des signes d'intolérance clinique avec une hypotension ou un état de choc, une asphyxie, une aphonie et un thorax immobile[8].

La confirmation diagnostique du PNO est apportée par la radiographie du thorax, examen indispensable qui permet une première estimation de la taille de l’épanchement. Le seul cliché validé est celui réalisé de face, debout et en inspiration, le cliché expiratoire n'apporte pas d'information suffisante pour être réalisé systématiquement [9]. En pratique on parle de pneumothorax important quand il est supérieur à 20% de la surface pulmonaire; ce qui correspond grossièrement à un décollement de 3cm à l'apex ou de 2cm en latéral. Le scanner thoracique et les explorations fonctionnelles respiratoires sont indiqués à distance, dans le cadre d'un bilan fonctionnel et anatomique à la recherche de lésions pulmonaires sous-jacentes [1, 9].

Les différentes méthodes thérapeutiques du PNO sont représentées essentiellement par le drainage thoracique conventionnel, l'exsufflation manuelle, la pose de drains de plus faible calibre type Pleurocath^®^ et les autres méthodes plus invasives telles que la vidéothoracoscopie et la chirurgie pleurale [9]. Cependant il n'y a pas de preuves scientifiques formelles ou de consensus en faveur de l'une ou l'autre des techniques [10, 11].

L'hospitalisation s'impose donc d'emblée en raison du risque de décompensation de la pathologie respiratoire sous-jacente. A l'inverse des PSP, l’évacuation de l'air dans les PSS fait d'emblée appel à la mise en place d'un drain thoracique car l'exsufflation simple à l'aiguille a un taux de succès beaucoup plus faible que dans les PSP [9, 10, 11]. Toutefois une décompression par exsufflation à l'aiguille s'impose en cas de détresse respiratoire en attendant la mise en place d'un drain pleural [12].

Le choix entre la mise du drain en siphonnage simple et la mise en aspiration douce d'emblée dépend essentiellement de la tolérance du pneumothorax. En effet, un PSS de petite taille peut suffire à faire décompenser une pathologie pulmonaire sous-jacente et donc justifier la mise en aspiration immédiate pour réexpandre le poumon[13].

L’évolution du PSS est assez souvent défavorable du fait de la décompensation respiratoire et infectieuse de l'insuffisance respiratoire chronique, sans compter les difficultés de ré-expension pulmonaire et du retrait des drains thoraciques [8].

Après un premier épisode de PNO, le taux de récidive varie entre 16 et 50%. Après un deuxième épisode ce taux est nettement plus élevé que 50% [14, 15]. La plupart des récidives surviennent entre 6 mois et 2 ans. Beaucoup de centres conseillent une pleurodèse dès la première récidive. Les effort physiques sont à déconseiller durant environ 3 semaines, le sport et la musiques avec des instruments à vent sont à déconseiller durant les 2 premiers mois environ. Ces conseils sont cependant uniquement basés sur le bon sens et n'ont pas fait l'objet d’étude scientifique [5].

## Conclusion

Malgré la multitude de travaux et de recommandations sur la prise en charge du pneumothorax, il n'y a toujours pas de consensus unanime codifiant la prise en charge de cette pathologie. Les décisions thérapeutiques sont discutées au cas par cas mais faisant le plus souvent appel à un geste de sauvetage dans le cas d'un PSS qui peut mettre en jeu le pronostic vital du malade.
